# Mitophagy in Alzheimer’s disease: Molecular defects and therapeutic approaches

**DOI:** 10.1038/s41380-022-01631-6

**Published:** 2022-06-03

**Authors:** Arnaud Mary, Fanny Eysert, Frédéric Checler, Mounia Chami

**Affiliations:** Institut of Molecular and Cellular Pharmacology, Laboratory of Excellence DistALZ, Université Côte d’Azur, INSERM, CNRS, Sophia-Antipolis, 06560 Valbonne, France

**Keywords:** Neuroscience, Cell biology

## Abstract

Mitochondrial dysfunctions are central players in Alzheimer’s disease (AD). In addition, impairments in mitophagy, the process of selective mitochondrial degradation by autophagy leading to a gradual accumulation of defective mitochondria, have also been reported to occur in AD. We provide an updated overview of the recent discoveries and advancements on mitophagic molecular dysfunctions in AD-derived fluids and cells as well as in AD brains. We discuss studies using AD cellular and animal models that have unraveled the contribution of relevant AD-related proteins (Tau, Aβ, APP-derived fragments and APOE) in mitophagy failure. In accordance with the important role of impaired mitophagy in AD, we report on various therapeutic strategies aiming at stimulating mitophagy in AD and we summarize the benefits of these potential therapeutic strategies in human clinical trials.

## Introduction

**Fig. 1:**
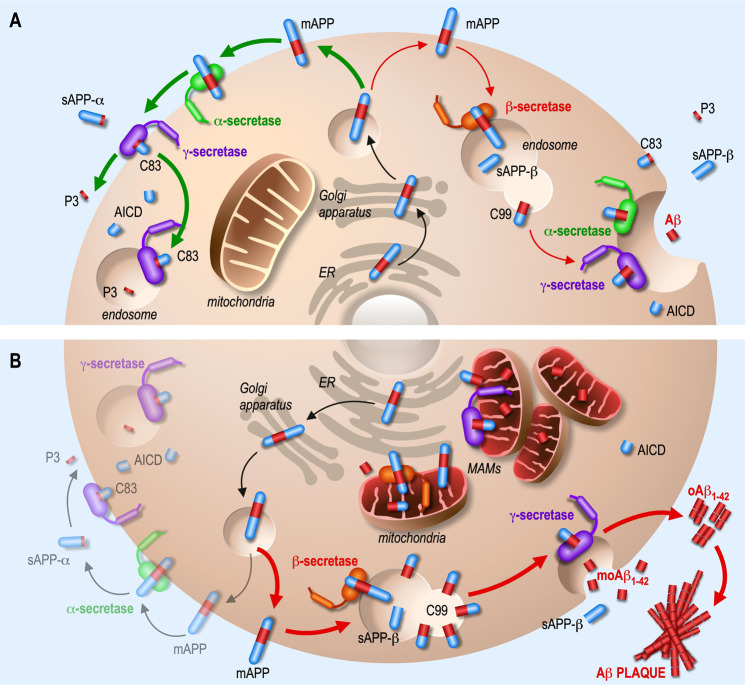
Amyloidogenic and non-amyloidogenic processing of APP. The amyloid precursor protein (APP) is synthesized in the endoplasmic reticulum (ER) and passes through the Golgi apparatus where it undergoes several post-translational modifications such as N- and O-glycosylations, sialylations, phosphorylations and sulfatations before reaching the plasma membrane (PM) in its mature form (mAPP). There, APP can be processed into two different pathways. **A** In physiological conditions, APP mainly follows the non-amyloidogenic pathway (green arrows), where it is cleaved by the α-secretase, producing secreted sAPPα fragment and membrane-anchored APP-CTF-α (C83), which is then cleaved by the γ-secretase generating secreted P3 fragment and APP intracellular domain (AICD) fragment. Under physiological conditions, APP is also marginally processed in the amyloidogenic pathway (red arrows), in which it is first internalized in endosomes and cleaved by the β-secretase generating sAPPβ fragment and membrane-anchored APP-CTF-β (C99). The latter is subsequently cleaved by the γ-secretase to generate Aβ and AICD (APP intracellular domain), or by the α-secretase to generate the C83 peptide. **B** In Alzheimer’s disease (AD), APP is mostly processed in the amyloidogenic pathway. It also accumulates and is processed in the intracellular compartments including the endoplasmic reticulum (ER) and mitochondria-associated membranes (MAMs), where secretases are also present. Secreted monomeric Aβ_1-42_ (moAβ_1-42_) peptide is prone to oligomerize (oAβ_1-42_) and generates extracellular amyloid plaques.

Alzheimer’s disease (AD) is a common and irreversible neurodegenerative disorder associated with age and characterized by two main histological hallmarks: the accumulation of amyloid beta (Aβ) aggregated in extracellular senile plaques, and the hyperphosphorylation of Tau protein (pTau) forming intracellular neurofibrillary tangles (NFTs) [1]. Major clinical symptoms of the disease are a decline of cognition and memory capacities attributed in sporadic AD (SAD) to environmental and/or genetic susceptibility factors, the first discovered being the *Apolipoprotein* (*APOE*) gene [2]. Familial AD (FAD) form is linked to monogenic mutations in the *Amyloid Precursor Protein* (*APP*) gene and in *Presenilin 1* (*PSEN1*) and *Presenilin 2* (*PSEN2*) genes, with the latter two encoding the catalytic core of the γ-secretase complex [3]. The discovery of AD-associated mutations led to the formulation of the amyloidogenic cascade hypothesis (Fig. 1) defining enhanced Aβ production and decreased clearance as the major triggers of AD development [4].

**Fig. 2:**
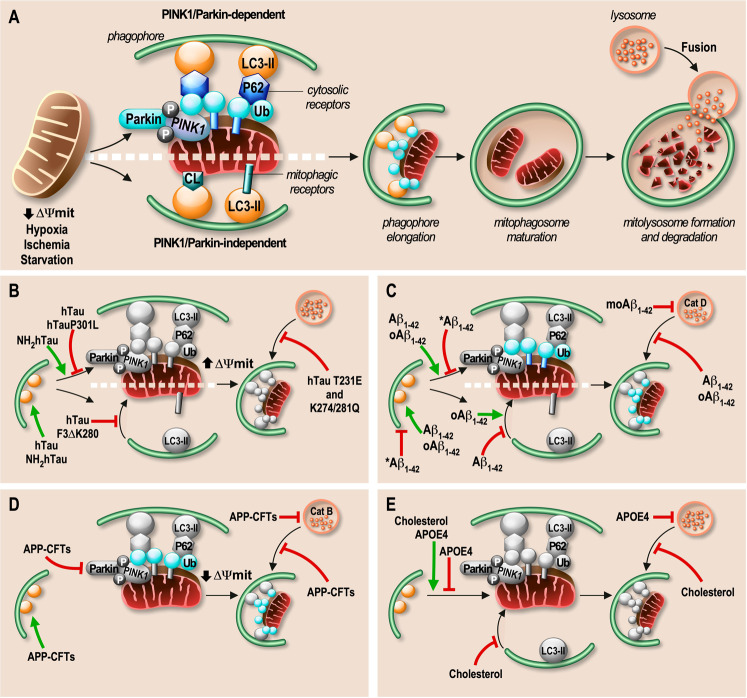
Physiological mitophagy processes and alterations linked to Tau, Aβ, APP-CTFs and APOE. **A** Following mitochondrial stress (i.e., mitochondria membrane (ΔΨmit) depolarization, or a general cellular stress (i.e. hypoxia, ischemia, starvation)), mitochondria undergo a specific degradation mechanism by mitophagy. The PINK1/Parkin-dependent mitophagy is driven by a decrease of ∆Ψmit, triggering the stabilization of PTEN-induced putative kinase 1 (PINK1) at the OMM and the recruitment of the E3 Ubiquitin ligase Parkin, generating poly-phospho-ubiquitinated chains (p-S65-Ub) on OMM proteins that act as a “eat-me” signal for damaged mitochondria. Cytosolic proteins that can act as mitophagy receptors (p62, OPTN, NDP52, NBR1, or TAX1BP1) will recognize and bind to p-S65-Ub decorated proteins and will recruit the phagosome through their interaction, via their LIR motif with LC3-II (the activated form of the microtubule-associated protein 1 A/1B light chain 3) present at the surface of the phagosome. Besides, in the PINK1/Parkin-independent mitophagy, the phagosome is recruited around mitochondria directly through LIR motif-containing OMM receptors (NIX/BNIP3L, BNIP3, FUNDC1, BCL2L13, FKBP8, DISC1, AMBRA1, or MCL-1) or through the recognition of cardiolipin (CL) exposed on the OMM. Once the phagophore is recruited, it engulfs damaged mitochondria to generate the mitophagosome. Then, lysosomes can fuse with mitophagosomes to form mitolysosomes and degrade damaged mitochondria by acidic hydrolases. **B**–**E** Mitophagic process is perturbed in AD by Tau species (**B**), Aβ species (**C**). APP-CTFs (**D**), and APOE4 isoform and/or high cholesterol levels (**E**). Proteins that are impacted in the mitophagy process are depicted in grey in (**B**–**E**) instead of being colored as in (**A**). Inhibitory functions are indicated by red arrows, and green arrows indicate PINK1/Parkin activation and phagophore formation or recruitment.

Mitochondria are self-replicating cellular organelles present in all human cells, except in erythrocytes. Mitochondria, through transporters and channels located on their outer membrane (OMM), ensures exchanges of calcium (Ca^2+^), metabolites, and lipids with the rest of the cytosol and with other organelles. This OMM is also the site of finely regulated events sensing mitochondrial-dependent cell death but also their specific degradation, dynamic fusion, fission and mobility. Mitochondria provide most of the cell energetic demands through the production of adenosine triphosphate (ATP) by mitochondrial oxidative phosphorylation (OXPHOS) occurring in the inner mitochondrial membrane (IMM) through five enzymatic complexes and two mobile electron carriers. The mitochondria matrix encompasses the mitochondrial genome (mitDNA for mitochondrial DNA) and is the site of the tricarboxylic acid cycle, the function of which is tightly linked with OXPHOS reactions and ensures the production of several important metabolites implicated in the biosynthesis of macromolecules such as nucleotides, lipids, and proteins. Beyond being the energy and metabolites providers, mitochondria generate reactive oxygen species (ROSmit), that act as pathophysiological sensors of a plethora of cell events including cell death, proliferation, differentiation and immunity. Physiological homeostasis of mitochondria is a balance between the biogenesis of functional mitochondria and autophagic degradation of dysfunctional or superfluous ones through a selective process referred to as mitophagy (Fig. 2A). During this clearing pathway, unhealthy mitochondria are engulfed by a double-membrane (phagophore) forming the mitophagosome, which will then fuse with a lysosome to form a mitophagolysosome. In this structure, lysosomal hydrolases will digest mitochondrion/mitochondria into small components that can then be recycled (Fig. 2A). Studies have identified two major types of mitophagy (PINK1/Parkin-dependent or independent) activated by a plethora of stimuli and implicating different proteins displaying a role of mitophagy receptors (Fig. 2A).

As neurons are sites of high-demanding energetic processes, their homeostasis is highly dependent on mitochondrial health and on the effectiveness of mitophagy. Neurons display the specificity that degradative lysosomes are predominantly located in the soma, thus requiring that distal damaged mitochondria/mitophagosome have to be transported in a retrograde manner back to the cell body for their degradation through dedicated transport machinery. Axonal mitochondrial quality control may also occur in a Parkin-dependent or -independent manner thereby providing rapid neuroprotection against stress conditions or early neurodegenerative disease stages [5, 6].

Mitochondrial impairment occurs prior to pathological stage associated with AD [7, 8] and thus may act as a “risk factor” of AD. Examination of results in animal and cellular models of AD mimicking familial forms of AD and in the brains of SAD patients, have then led to the hypothesis that impaired mitophagy is an important aspect of the disease, and could lie both upstream and downstream of Aβ and Tau in a vicious circle ultimately causing synaptic dysfunctions and cognitive deficits [9].

This review discusses studies examining the status of mitophagy in AD patients, as well as in various in vitro and in vivo AD models. We provide insights into the specific impact of different actors implicated in AD etiology, including Tau, APOE4, presenilin 1 (PS1), APP-derived fragments (Aβ and APP C-terminal fragments: APP-CTFs) on mitophagy processes (Fig. 2B). We also describe the different strategies (genetic, pharmacological and lifestyle) aimed at promoting the removal of excess dysfunctional mitochondria by mitophagy, and highlight their beneficial effects on various histological, biochemical, and behavioral/cognitive phenotypes associated with AD.

## Evidence for impaired mitophagy in ad patients AD brains

Impaired mitophagic degradation in AD has been suspected since the early 2000s. This was suggested by the accumulation of mitDNA and proteins in the cytoplasm, as well as in autophagic vacuoles (AVs) in AD neurons featuring increased oxidative damages [10]. AD brains analyses unraveled increased levels of mitochondrial proteins COX IV and TOMM20, and elevated ratio of mitDNA/nuclear DNA [11]. Other results then supported reduced mitophagic degradation in patient brains with high levels of total and pTau protein, suggesting a participation of Tau protein in this phenotype. Molecularly, an increase of PINK1 at early AD stages (Braak stage II-III) and of Parkin at a later stage (Braak stage VI), alongside with an increase of mitochondrial content markers at both early and late stages were reported in SAD hippocampi [12]. This could suggest a reduced mitophagic flux linked to a defect in the initiation of the PINK1/Parkin cascade. This has been supported by another study reporting decreased mRNA and protein levels of PINK1 in hippocampi at late-stage AD (Braak stage V-VI) [13]. In addition, Martín-Maestro et al. also observed in AD-affected brains a downregulation of the expression of various proteins known to participate to autophagy and mitophagy processes, namely Optineurin (OPTN), ATG5, ATG12, Beclin-1 (Bcl-1), PI3K class III, ULK1, AMBRA1, BNIP3, BNIP3L, FUNDC1, VDAC1, and VCP/P97 [14]. Accordingly, a reduction of the expression of Bcl-1, ATG12, BNIP3, and PINK1, a decrease of p-S65-Ub, and an increase of the levels of LC3-II and p62/SQSTM1 (referred hereafter as p62) were also observed in the brains of APOEε4 heterozygote patients [15]. This was thought to be associated with an elevation of the inactive form of FOXO3a (Forkhead box O3a) transcription factor sequestered in the cytosol [15]. In 2019, Fang et al. showed that basal levels of mitophagy are reduced by 30–50% in post-mortem hippocampal brain samples from AD patients compared with sex and age-matched cognitive normal ones [16]. This mitophagy reduction was characterized by the accumulation of structurally and functionally damaged mitochondria (reduced size, disorganized cristae and low ATP production), and by an impairment of the initiation steps of the mitophagy process (reduced activated LC3 recruitment to mitochondria, and a dysfunctional AMP-activated protein kinase (AMPK) cascade, characterized by the phosphorylation of AMPK and the inhibition of its targets ULK1 and TBK1) [16]. Accordingly, these observations were consolidated by the report of a defective fusion of mitophagosomes with lysosomes [16] and an accumulation of damaged mitochondria in AVs [17] in AD brains. Interestingly, our laboratory also reported defective mitophagy in a cohort of human SAD brains (Braak stage IV-VI) supported by an increase of LC3-II/I ratio and of p62 and a reduction of PINK1 and of Parkin levels in mitochondria-enriched fraction [18]. It is worth noting that while all mitophagic markers variations correlated with APP-CTFs accumulation, Aβ and pTau levels only correlated with reduced Parkin and increased LC3-II/I ratio respectively, suggesting a stronger link of mitophagy failure with APP-CTFs accumulation in human AD brains [18].

The retrograde transport of distal damaged mitochondria towards the neuron soma where they should be degraded in mitolysosomes is also impaired in AD patients. Indeed, Wang et al. reported that DISC1, a protein regulating axonal mitochondria trafficking, is less abundant in AD prefrontal cortices [19]. Strikingly, they also demonstrated a novel role for DISC1 as a mitophagy receptor present in both the OMM and the IMM, that recruits phagophores to damaged mitochondria by binding to LC3-II through its LIR (LC3-interacting region) motif [19].

Reduced activity of mitochondrial complexes I, II and V occurs as early as AD stages I-II in the entorhinal cortex (EC) [8], suggesting that mitochondrial dysfunctions and mitophagy failure occurs primarily in vulnerable brain region. These alterations are likely to be amplified by Aβ and contribute to pTau accumulation during the prodromal stages of AD [7].

### AD patient-derived cells and fluids

Skin fibroblasts from SAD patients also display dysfunctional autophagy and mitophagy illustrated by reduced formation of AVs, lower number of lysosomes and accumulation of TOMM20 mitochondrial marker [12]. Parkin levels are also reduced in mitochondria-enriched fraction, even upon mitophagy stimulation with the mitochondrial membrane uncoupler (CCCP treatment), known to trigger PINK1 stabilization to the mitochondria and, thereby, enhancing Parkin recruitment and phosphorylation [12]. Other studies demonstrated that SAD fibroblasts feature “aged mitochondria” and harbor an impaired capacity of degrading them [14, 20]. This phenotype has been further supported by using MitoTimer probe that allows to follow the maturation of mitochondria by recording a shift of the emission wavelength. This demonstrated that fibroblasts from healthy individuals display a spatial maturation gradient, with a localization of young mitochondria at the periphery and old mitochondria surrounding the nucleus. Strikingly, SAD fibroblasts do not display such distribution, but rather showed altered mitochondria transport to the degradation site and an accumulation of dysfunctional mitochondria [20]. Martín-Maestro et al. additionally demonstrated impaired autophagy and mitophagy in fibroblasts of a FAD patient harboring a PS1(A246E) mutation and in induced pluripotent stem cells (iPSC)-derived neurons [21]. Both models showed enhanced LC3-II levels and impaired lysosomal degradation capacities reflected by accumulations of p62 and TOMM20. However, unlike SAD fibroblasts, Parkin levels were increased in FAD PS1(A246E) fibroblasts, compared with non-isogenic controls [21]. Yet, these observations have to be further confirmed using isogenic controls. Other studies also report lysosomal defects in human FAD fibroblasts with other *PS1* mutations linked to lysosomal alkalization, reduced cathepsin D activity and overall lowered macroautophagic degradation [22, 23]. iPSC-derived neural stem cells (iNSC) Knocked-in (KI) for PS1(M146L) also harbor a downregulation of autophagy characterized by decreased LC3 level and ULK1 activation, accumulation of p62 and reduced TFEB (transcription factor EB, a master gene for lysososmal biogenesis) expression and LAMP1 levels reflecting decreased lysosomal activity [24]. These observations were accompanied by an increased expression of PINK1 and Parkin, enhanced Parkin recruitment to the mitochondria, and an impairment of the recruitment of the phagophore. This would suggest an initiation of mitophagy process and a blockade at the stage of mitophagosome formation. In fact, authors reported an accumulation of mitochondria revealed by an elevated number of mitDNA copies and enhanced TOMM20 level [24]. Interestingly, autophagy failure was also reported in iPSC-derived cortical neurons obtained from APP(V717L) and APOEε4 patients characterized by reduced levels of Bcl-1 and LC3-II and of the number of autophagosomes and autolysosomes [16]. These cells also showed impaired mitophagy, since phosphorylations of TBK1 and ULK1 were reduced as well as the levels of several mitophagic proteins [16] (Table 1). Neuroblastoma SH-SY5Y cybrid cells depleted from their mitochondrial content and complemented by mitochondria from AD patient platelets show altered mitochondrial structure and activity (reduced ATP and enhanced ROSmit productions) associated with impaired mitophagy likely linked to PINK1 downregulation [25].

**Table 1:** Mitophagic failure molecular signatures in AD patient brains, fluids and cells.

	Experimental approaches/methods	Molecular modifications	Expected phenotypic manifestations	Refs.
Hippocampus Braak stage II-III	WB & RT-qPCR	↑ TOMM20 protein ↑ Mitochondrial mRNA 16S/MT-NRN2	↑ Mitochondrial mass	[12]
		↑ PINK1 and PINK1_52_ proteins ↔ Parkin protein	↓ PINK1/Parkin activation	
Hippocampus & frontal cortex Braak stage V-VI	WB & RT-qPCR	↑ TOMM20 protein ↑ Mitochondrial mRNA 16S/MT-NRN2	↑ Mitochondrial mass	
		↔ PINK1 and PINK1_52_ proteins ↑ Parkin protein	↓ PINK1/Parkin activation	
ND	RT-qPCR	↓ PINK1 and Parkin mRNA	↓ PINK1/Parkin activation	
Hippocampus & frontal cortex Braak stage II-III & V-VI	WB	↓ Cytosolic Parkin protein	↓ PINK1/Parkin activation	[17]
ND	TEM	↑ Number of damaged mitochondria		
	WB	↑ Parkin protein	Parkin activation	
		↔ p62, TOMM20 and HSP60 proteins	↓ lysosomal degradation	
	TEM	↑ Number of mitochondria in autophagic vesicles		
	WB	↑ LC3-II/I protein ratio	↑ Phagophore recruitment	
Hippocampus & frontal cortex Braak stage V-VI	RT-qPCR	↓ PINK1 mRNA and protein	↓ PINK1/Parkin activation	[13]
	ELISA	↑ p-S65-Ub protein	↑ Mitochondria tagged for degradation	[28]
ND	TEM	↑ Number of damaged mitochondria		[16]
	WB	↑ pT172 AMPK protein ↓ pS555 ULK1 and pS172 TBK1 proteins	↓ Mitophagy initiation	
		↓ LC3-II protein	↓ Phagophores number	
	IHC	↓ TOMM20 – LAMP2 colocalization	↓ Mitolysosomes formation	
	WB	↓ DISC1 protein	↓ PINK1/Parkin-independent mitophagy	[19]
	Microarray analysis	↓ NIX, BNIP3 and FUNDC1 mRNA	↓ PINK1/Parkin-dependent mitophagy	[14]
		↓ OPTN, VDAC1 and VCP/p97 mRNA	↓ PINK1/Parkin-independent mitophagy	
	WB of Mit fraction	↓ PINK1 and Parkin protein	↓ PINK1/Parkin activation	[18]
		↑ p62 protein	↓ Lysosomal degradation	
		↑ LC3-II/I protein ratio	↑ Phagophore recruitment	
Bearers of APOEε4 allele	WB	↓ FOXO3a protein ↑ pS253 / tot FOXO3a ratio	↓ FOXO3a activity	[15]
		↓ Bcl-1 and ATG12 proteins	↓ Phagosomes formation	
		↓ PINK1 protein ↓ p-S65-Ub protein	↓ PINK1/Parkin activation	
		↔ p62 protein	↓ Lysosomal degradation	
Plasma	RT-qPCR	↑ PINK1 & LC3 mRNA ↓ Parkin mRNA	ND	[27]
Serum	ELISA	↓ Parkin & ATG5 proteins	ND	[26]
SAD fibroblasts	WB (Mit fraction ± CCCP	↑ TOMM20 protein ↓ Parkin protein	↑ Mitochondrial mass ↓ PINK1/Parkin activation	[12]
	IF ± CCCP	↓ Parkin & SCaMC-1 colocalization	↓ Parkin recruitment to mitochondria	
	WB	↓ LAMP1 protein	Defective lysosomal function	
	MitoTimer probe	↓ Mitochondria maturation	Accumulation of dysfunctional mitochondria	[20]
	WB	↓ PINK1 protein ↑ LC3-II/ I ratio	↓ PINK1/Parkin activation ↑ Phagophores number	
	IF & MTG probe	↔ p62 protein & ↔ mitochondrial mass	↓ Degradation	
PS1 (A246E) fibroblasts	IF & WB	↑ TOMM20 protein ↑ Parkin protein	↑ Mitochondrial mass ↑ PINK1/Parkin activation	[21]
	IF ± CCCP	↑ Parkin & TOMM20 colocalization	↑ Parkin recruitment to mitochondria	
	IF ± CCCP	↑ LC3 & TOMM20 colocalization	↑ Phagophore recruitment	
	LysoTracker probe ± CCCP & Baf	↓ Lysosomes acidification	↓ Lysosomal degradation	
PS1 (A246E) iPSC-differentiated neurons	IF & WB	↑ TOMM20 protein ↑ PINK1 & Parkin proteins	↑ Mitochondrial mass ↑ PINK1/Parkin activation	
	IF ± CCCP	↑ Parkin & TOMM20 colocalization	↑ Parkin recruitment to mitochondria	
	WB	↓ Pro-cathepsin B activation	↓ Lysosomal degradation	
APP (V717L) & APOEε4/ε4 iPSC-differentiated neurons	WB	↓ Bcl-1 protein & pS555 ULK1 and pS172 TBK1 proteins ↓ PINK1 protein ↓ AMBRA1, BCL2L13 & FUNDC1 proteins	↓ Mitophagy initiation ↓ PINK1/Parkin initiation ↓ PINK1/Parkin-independent mitophagy activation	[16]
KI PS1 (M146L) iPSC-differentiated neural stem cells	WB ± CCCP	↑ TOMM20 protein ↑ PINK1 & Parkin proteins	↑ Mitochondrial mass ↑ PINK1/Parkin activation	[24]
	IF ± CCCP	↑ PINK1 & Parkin proteins ↑ Parkin & TOMM20 colocalization	Parkin recruitment to mitochondria ↓ Phagophore recruitment	
	RT-qPCR and WB	↓ LC3 & TOMM20 colocalization ↓ TFEB & LAMP1 mRNA & proteins	↓ Lysosomal degradation	
Neuronal cybrids	WB	↓ PINK1 protein	↓ PINK1/Parkin activation	[25]

Phenotype modifications and/or levels of mRNA or proteins are depicted as ↑ (increased), ↓ (decreased), or ↔ (unchanged) as compared to control individual (brain samples, blood, serum and cells). *ND* Not determined, *iPSC* Induced pluripotent stem cells, *iNSC* Induced neural stem cells, *FAD* Familial Alzheimer’s disease, *SAD* Sporadic Alzheimer’s disease, *PS1/2* Presenilin 1/2, *KI* Knock in, *WB* Western blot, *TEM* Transmission electron microscopy, *IHC* Immunohistochemistry, *IF* Immunofluorescence, *Baf* Bafilomycin, *CCCP* Carbonyl cyanide m-chlorophenylhydrazone, *Mit fraction* Mitochondrial fraction, *MTG* Mitotracker green probe.

Interestingly, a molecular signature of impaired mitophagy was also reported in the peripheral fluids of AD patients as evidenced by a reduction of the autophagic ATG5 factor and of Parkin levels in the serum [26], and a decrease of Parkin alongside with an increase of PINK1 and LC3 mRNA levels in peripheral blood [27]. From these studies have emerged the idea to use mitophagy molecular actors as peripheral biomarkers of AD. Conveniently, Watzlawik et al. have recently developed a sensitive sandwich ELISA allowing the detection of p-S65-Ub levels in different tissues [28] and reported elevated p-S65-Ub levels in the frontal cortex of AD patients (late Braak stage V-VI) compared to control individuals [28].

## Evidence for impaired mitophagy in ad models

Studies in post-mortem human brains evidenced alterations of the expression (mRNA or proteins) of several key actors known to participate to autophagy and or mitophagy processes (Table 1). However, these studies have produced only correlative observations that link the regulation of the expression of these markers with disease stages. The study of human-derived cells has then unraveled the potential implication of molecular actors associated with SAD and FAD cases (Table 1). Nonetheless, the specific implication of AD-related proteins, namely pTau and its truncated forms, Aβ peptides and other APP-derived fragments and APOE genotype in mitophagy process was formally demonstrated in AD cellular and animal models. Studies in cells helped to specifically monitor autophagy/mitophagy flux quantifying the dynamics of clearance of specific mitochondrial markers in presence or absence of autophagy inhibitors and/or using mitophagic reporters.

### Tau protein

Studies of the impact of Tau protein on mitophagy has produced some results that could appear paradoxical. It was reported that the neurotoxic 20–22 kDa NH_2_-Tau fragment (NH_2_hTau), mapping between 26 and 230 amino acids of the longest human tau isoform, harmfully stimulates mitochondrial degradation by mitophagy [29]. The expression of NH_2_hTau in mature hippocampal primary neurons triggers synaptic alterations, alters mitochondria structure and enhances mitophagic flux as evidenced by treatment with inhibitors of both fusion of autophagosome with lysosome and lysosomal protein degradation [29]. Further, pharmacological or genetic inhibition of mitophagy prevented NH2hTau-mediated injuries [30]. Authors postulated that the accumulation of NH_2_hTau fragment may contribute to synaptic injuries in AD by exacerbating mitophagy activity. However, other studies rather reported an inhibitory effect on mitophagy triggered by full-length human wild-type Tau accumulation [11, 31]. Hu et al. showed that hTau overexpression leads to an accumulation of total and pTau in the OMM fraction, and increases ∆Ψmit, thus preventing the stabilization of PINK1 into mitochondria and the subsequent Parkin recruitment [11]. In addition, Cummings et al. found that the overexpression of both hTau and hTau(P301L) (a frontotemporal dementia-associated mutation) inhibited mitophagy in neuroblastoma cells and in *C. elegans* model [31]. Authors noticed that mitophagy blockade was not due to a change in the ∆Ψmit but to an aberrant interaction between the projection domain of Tau protein and Parkin, impairing its translocation to damaged mitochondria [31]. The *C. elegans* BR5270 strain expressing the pro-aggregant F3ΔK280 hTau fragment in neurons, also shows dysfunctional mitochondria and a reduced number of mitophagy events [16]. Interestingly, to avoid any inaccurate conclusion linked to overexpression strategies, the group of Nehrke, K. studied a transgenic *C. elegans* model expressing a single-copy of hTau [32]. While single-copy expression of hTau did not elicit overt pathological phenotypes, mimetics of AD-related post-translational modifications (PTM) (T231E and K274/281Q) of Tau exhibited age-dependent behavioral and neuronal morphological abnormalities. The same study reported that PTM mutants lacked the ability to engage in neuronal mitophagy in response to mitochondrial stress induced by paraquat [32].

Together, these results demonstrated that mitophagy impairment may be linked to Tau pathogenesis and stressed that distinct conclusions could be drawn according to the nature of the Tau protein analyzed and to the model and methods used to monitor mitophagy (Fig. 2B and Table 2).

**Table 2:** Involvement of Tau, Aβ, APP-CTFs, and AD risk factors in mitophagy failure in vitro and in vivo.

	Models	Experimental approaches/methods	Molecular mechanisms	Expected/reported phenotypes	Refs.
**Tau species**					
NH_2_hTau	I^ary^ hippocampal neurons from rats	RT-PCR	↓ mitDNA (mtND2)/gDNA (Htert) ratio	↑ Mitochondrial degradation	[29]
		WB	↓ TOMM20, VDAC1, TIMM23, CytC, ATPB & Mn-SODII proteins		
			↑ LC3-II protein	↑ Phagophores number	
			↑ PINK1 protein & ↑ Parkin protein in mitochondria	↑ PINK1/Parkin activation	
			↓ p62 protein	↑ Lysosomal degradation	
	Tg2576 mice (6 months)	WB (Mit fraction from purified synaptosomes)	↑ Parkin protein	↑ Parkin recruitment to mitochondria	[30]
hTau	HEK293T cells	WB	↑ TOMM20 & COX IV proteins	↑ Mitochondrial mass	[11]
		RT-PCR	↑ mitDNA (Atp-6)/gDNA (RpI13) ratio		
		WB (Mit fraction)	↓ PINK1 & Parkin proteins ↑ p62 protein	↓ PINK1/Parkin activation ↓ Lysosomal degradation	
	Mice I^ary^ hippo neurons	WB	↑ LC3-II protein	↑ Phagophores number	
hTau (P301L)	N2a cells	IF (Parkin transfection ± CCCP) (Mit fraction)	↓ Parkin protein	↓ Parkin activation	[31]
		Co-IP & PLA	↑ hTau (P301L)-Parkin interaction		
		IF (Parkin transfection ± CCCP)	↓ Mitochondria ubiquitination	↓ Mitochondria tagged from degradation	
	*C. elegans* (CK12)	IHC & crossing with mito-Rosella worms	↓ Mitochondria degradation in mitolysosomes	↓ Lysosomal degradation	
hTau F3ΔK280	*C. elegans* *(*BR5270)	IHC ± PQT	↓ LC3-BNIP3/NIX colocalization	↓ Phagophores recruitment	[16]
hTau T231E & K274/281Q	*C. elegans*	IHC ± PQT	↓ Mitolysosomes number	↓ Mitolysosomes formation	[32]
**Aβ species**					
Aβ_1-42_	PC12 cells (5 µM, 24 h)	WB & RT-qPCR	↑ Bcl-1 & ↑ Parkin mRNA & protein	↑ Mitophagy initiation	[34]
		WB	↑ LC3 protein & LC3-II/I ratio	↑ Phagophores number	
	PC12 cells (7 µM, 12 h)	WB	↓ Bcl-1 protein	↓ Mitophagy initiation	[33]
			↓ PINK1 & Parkin proteins	↓ PINK1/Parkin activation	
			↓ LC3-II protein	↓ Phagophores number	
			↑ p62 protein	↓ Lysosomal degradation	
	*C. elegans* (CL2355)	IHC ± PQT	↓ LC3-BNIP3/NIX colocalization	↓ Phagophores recruitment	[16]
	CA1 injected rats	IHC & WB	↓ Bcl-1 protein	↓ Mitophagy initiation	[37]
			↓ PINK1 & Parkin proteins	↓ PINK1/Parkin activation	
			↑ p62 protein	↓ Lysosomal degradation	
	Mouse I^ary^ hippo neurons (5 µM)	WB	↑ Bcl-1 protein	↑ Mitophagy initiation	[35]
			↑ LC3-II protein	↑ Phagophores number	
			↑ PINK1 & Parkin proteins ↑ Parkin protein in mitochondria	↑ PINK1/Parkin activation	
		IF & mRFP-GFP-LC3 probe	↓ Mitolysosomes number	↓ Mitophagy	
oAβ_1-42_	HEK293T cells (10 µM)	WB	↑ mitochondria number	Mitochondria accumulation	[36]
		WB (Mit fraction)	↑ Parkin protein	Parkin activation	
			↑ LC3-II protein	↑ Phagophores recruitment	
		TEM	↑ Mitophagosomes number	Mitophagosomes accumulation	
		WB (Mit fraction)	↑ p62 protein	↓ Lysosomal degradation	
mAβ_1-42_	SK-N-BE cells (1 µM)	WB	↑ Bcl-1 protein	↓ Mitophagy initiation	[39]
		Co-IP	↓ Bcl-1 & Bcl-2 protein complex		
		WB	↑ LC3-II protein	↑ Phagophores number	
		IF & GFP-RFP-LC3 probe	↑ Autophagosomes number ↑ Autolysosomes number	Autophagosomes accumulation	
		WB	↑ p62 protein		
		Enzymatic assay	↓ CTSD activity	↓ Lysosomal degradation	
**APP-CTFs**					
AICD	HEK293T	WB & RT-qPCR	↑ PINK1 mRNA & protein	↑ PINK1/Parkin activation	[41]
	SH-SY5Y	WB	↑ LC3-II protein	↑ Phagophores number	
			↓ P62, TOMM20 & TIMM23 proteins	↑ Lysosomal degradation	
C99 and C83	SH-SY5Y APPswe ± γ-sec inhibitor	WB	↑ LC3-II protein	↑ Phagophores number	[45]
			↓ Mature CTSB protein	↓ Lysosomal degradation	
		Enzymatic assay	↓ CTSB activity		
	3xTgAD mice ± γ-sec inhibitor	WB	↑ LC3-II protein	↑ Phagophores number	
		TEM	↑ Autophagic vesicles number		
		WB	↑ p62 protein	↓ Lysosomal degradation	
	AAV-C99 mice	IHC	↑ LAMP1 puncta number & size	Lysosomal failure	
	AAV-C99 mice ± γ-sec inhibitor	WB	↑ LC3-II protein	↑ Phagophores number	
		IHC	↑ CTSB puncta number & size	Lysosomal failure	
	SH-SY5Y APPswe	WB (Mit fraction)	↑ PINK1 & Parkin proteins	↑ PINK1/Parkin activation	[18]
		WB (Mit fraction)	↑ LC3-II protein	↑ Phagophores number	
		GFP- LC3 probe	LC3 puncta		
		IF	↑ HSP60- LC3 colocalization	↑ Mitophagosomes number	
			↓ mitochondria-LAMP1 colocalization	↓ Mitolysosomes number	
		WB (Mit fraction)	↔ p62 protein	↓ Lysosomal degradation	
			↑ TOMM20, TIMM23, HSP60 & HSP10 proteins		
	SH-SY5Y APPswe ± γ-sec inhibitor	TEM	↑ Mitochondria number with damaged cristae	Damaged mitochondria accumulation	
		WB (Mit fraction)	↔ PINK1 & Parkin proteins	↓ PINK1/Parkin activation	
			↑ LC3-II/I protein ratio	↑ Phagophores number	
		Cox8-EGFP-mCherry probe	↔ Cells number containing mitolysosomes	↔ Mitolysosomes number	
	SH-SY5Y C99	WB (Mit fraction)	↔ PINK1 & Parkin proteins	↓ PINK1/Parkin activation	
			↔ LC3-II/I protein ratio	↔ Phagophores number	
			↑ p62, TOMM20 & HSP10 proteins	↓ Lysosomal degradation	
	AAV-C99 mice	WB (Mit fraction)	↑ LC3-II protein	↑ Phagophores number	
			↔ p62, TOMM20 & HSP10 proteins	↓ Lysosomal degradation	
	Young 3xTgAD mice ± γ-sec inhibitor	TEM	↑ Mitochondria with damaged cristae	Damaged mitochondria accumulation	
		WB (Mit fraction)	↔ PINK1 protein ↑ LC3-II/I protein ratio	↓ PINK1/Parkin activation	
			↔ p62 protein	↑ Phagophores number	
			↑ TIMM23 & HSP10 proteins	↓ Lysosomal degradation	
	Old 3xTgAD & 2xTgAD mice	TEM	↑ Mitochondria with damaged cristae	Damaged mitochondria accumulation	
		WB (Mit fraction)	↑ PINK1 protein	↑ PINK1/Parkin activation	
			↑ LC3-II/I protein ratio	↑ Phagophores number	
			↓ p62 & TIMM23 proteins	↑ Lysosomal degradation	
	iNSCs from FAD patient (PS1 C737A)-derived fibroblasts	WB ± Baf	↑ LC3-II protein & LC3-II/I ratio	↑ Phagophores recruitment	[57]
			↑ p62 protein	↓ Lysosomal degradation	
		IF	↓ LC3 & LAMP1 colocalization	↓ Autolysosomes number	
		WB ± γ-sec inhibitor	↑ LC3-II protein		
			↑ PINK1 & Parkin proteins	↑ PINK1/Parkin activation	
			↑ p62 protein		
			↑ HSP60 protein		
		IF ± γ-sec inhibitor	↓ MitoTracker & LAMP1 colocalization	↓ Lysosomal degradation	
				↓ Mitolysosomes number	
	PS1/2 KO iNSCs	WB	↑ PINK1 & Parkin proteins	↑ PINK1/Parkin activation	
			↑ LC3-II protein	↑ Phagophore recruitment	
			↑ p62 protein	↓ Lysosomal degradation	
		IF	↓ LC3 & LAMP1 colocalization	↓ Autolysosomes number	
			↑ HSP60 & p62, Parkin or Ubiquitin colocalization	↑ Mitophagy initiation	
**Risk factors**					
APOE4	T98G cells	Proteomic analysis	↑ TOMM5, ATP5B, CS & SDHA aggregation	Mitochondria accumulation	[58]
		DNA pulldown assay	Competition for TFEB CLEAR motif	↓ Expression of mitophagy actors	
		RT-PCR	↓ MAP1LC3B, p62/SQSTM1 & LAMP2 transcription		
	Hippocampus from hAPOEε4/ε4 mice	IHC & WB	↑ TOMM40 & COX I proteins	Damaged mitochondria accumulation	[60]
		TEM	↑ Mitochondrial length ↓ Mitochondria cristae density		
		WB	↓ PINK1_52_ protein	↑ PINK1/Parkin activation	
		IHC & WB	↑ Parkin protein		
	Astrocytes from hAPOEε4/ε4 mice	WB	↔ p62 protein ↑ TOMM40 & TOMM20 proteins	↓ Lysosomal degradation Mitochondria accumulation	[61]
		RT-qPCR & WB (Mit fraction)	↑ Parkin mRNA & protein in mitochondria	↑ PINK1/Parkin activation	
		Co-IP	↑ Ubiquinated Parkin	↓ Parkin activity	
		WB ± CQ (Mit fraction)	↓ LC3-II/I protein ratio	↓ Mitophagosomes number	
		IF & Mito-GFP ± CCCP	↓ Mito-GFP and LC3 colocalization	↓ Mitophagosomes number	
		TEM ± CCCP	↑ PINK1 & ↓ PINK1_52_ protein	↓ Lysosomal degradation	
		LC3-EGFP-mRFP probe ± CCCP	↓ Mitolysosomes number ↓ Autolysosomes number	↓ Lysosomal degradation	
		WB ± CCCP±CQ	↓ LC3-II protein		
High cholesterol	SH-SY5Y cells treated with oAβ_1-42_	IF	↑ CYC- LC3-II colocalization ↓ CYC-LAMP2 colocalization	↑ Mitophagosomes number ↓ Mitolysosomes number	[63]
	APP/PS1 mice, SREBF2 mice	Digital PCR	↑ mitDNA copy number	Damaged mitochondria accumulation	
		WB (Mit fraction) Phos-Tag SDS-PAGE (Mit fraction)	↑ poly-Ubiquinated K63 ↑ PINK1 & Parkin proteins ↑ Phosphorylated PINK1	↑ PINK1/Parkin activation	
		IHC	↓ OPTN translocation to mitochondria	↓ Phagophore recruitment	

Phenotype modifications and/or levels of proteins are depicted as ↑ (increased), ↓ (decreased), or ↔ (unchanged) as compared to respective control models. *∆Ψm* Mitochondrial membrane potential. *CA3* Cornu ammonis, *C. elegans* *Caenorhabditis elegans*. *oAβ* Oligomeric β amyloid peptide, *mAβ* Monomeric β amyloid peptide, *I*^*ary*^ Primary, *Hippo* Hippocampal, *mitDNA* Mitochondrial DNA, *gDNA* Genomic DNA, *CTSD* Cathepsin D, *CTSB* Cathepsin B, *TEM* Transmission electron microscopy, *WB* Western blot, *IF* Immunofluorescence, *IHC* Immunohistochemistry, *Co-IP* Co-immunoprecipitation, *PLA* Proximity ligation assay, *Mit fraction* Mitochondrial fraction, *CCCP* Carbonyl cyanide m-chlorophenylhydrazone, *PQT* Paraquat, *γ-sec* γ-secretase, *Mito-GFP* GFP targeted to mitochondria, mRFP-GFP-LC3 and GFP-RFP-LC3 probes: two tandem fluorescent-tagged LC3 probe monitoring autophagic flux based on different pH stability of EGFP and mRFP, Cox8-EGFP-mCherry probe: a tandem fluorescent-tagged mitochondrial targeting sequence of inner membrane protein COX8 (cytochrome c oxidase subunit 8), *3xTgAD* mice (APPswe, TauP301L & PS1 M146V), *2xTgAD* mice (APPswe, TauP301L & PS1 WT), *CQ* Chloroquine.

### Aβ peptides

Again, studies on the influence of Aβ peptides on mitophagy raised divergent conclusions. A first study reported that a 12 h exogenous application of Aβ_1–42_ peptide at 7 µM on rat pheochromocytoma cells (PC12) triggered an impaired mitophagy characterized by reduced levels of PINK1, Parkin, Bcl-1 and LC3-II/I ratio, as well as an accumulation of p62 [33]. Conversely, another study reported that a longer (24 h) exogenous application of Aβ_1–42_ peptide at an almost similar concentration (5 µM) rather induced an increase of Parkin, Bcl-1, and LC3-II/I in PC12 cells [34]. Corroborating the latter study, an increase in PINK1, Parkin, LC3-II, and Bcl-1 expressions associated with mitochondrial dysfunction, reflected by reduced ∆Ψmit and of ATP level and enhanced ROSmit production, was observed in murine embryonic hippocampal neurons after 72 h of treatment [35]. Authors also demonstrated a blockade of the fusion of phagophores with lysosomes, evidenced by a reduction of GFP signal quenching of the fluorescent mitochondrial RFP-GFP-LC3 probe [35]. These results point toward either a defective elimination of dysfunctional mitochondria by mitophagy linked to Aβ_1–42_, characterized by a defective initiation of mitophagy or alternatively, toward a normal mitophagy initiation followed by a blockade at later steps of the process. Oligomeric Aβ_1–42_ (oAβ_1–42_) treatment of human non-neuronal cells (HEK293T) also caused an induction followed by a blockade of mitophagy process, as demonstrated by an increase of Parkin levels as well as of the LC3-II/I ratio and an accumulation of p62 in the mitochondrial fraction [36]. The conclusion of a defective mitophagy linked to oAβ_1–42_ application was strengthened by the observation of an accumulation of both autophagosomes and mitochondria [36]. These data were validated in vivo. Indeed, intracerebroventricular injection of Aβ_1–42_ in rats triggers reduced levels of PINK1, Parkin, and Bcl-1 and an accumulation of p62 [37]. Similarly, reduced mitophagy activity was also reported in an Aβ_1–42_-neuron-expressing *C. elegans* (CL2355) [16]. Interestingly, PINK1 overexpression reduces APP and Aβ_1–42_ levels in vivo, thereby demonstrating the ability of PINK1 to rescue Aβ_1–42_-associated defects [13]. In mice expressing hAPPswe/Ind, Tammineni et al. found that Aβ peptide and soluble oAβ interact with AVs, causing their impaired axonal retrograde transport and their accumulation in neuron terminals [38]. Guglielmotto and collaborators reported that the monomeric Aβ_1–42_ (moAβ) also triggers a blockade of the autophagy flux in differentiated human neuroblastoma cells (SK-N-BE). This was evidenced by the accumulation of p62 and of autophagosomes and by reduced lysosomal activity [39] (Fig. 2C and Table 2).

### APP-CTFs

The APP Intracellular Domain (AICD) generated by the cleavage of C83 or C99 fragments by γ-secretase (Fig. 1) transcriptionally controls the expression of key genes implicated in AD [40]. Our team recently reported that AICD controls mitophagy through the transcriptional activation of *PINK1* [41]. The study depicted the molecular cascade linking PINK1 to mitophagy process by demonstrating that PS1, but not PS2, triggers the transactivation of *PINK1* promoter in a FOXO3a-dependent manner and that enhanced mRNA and protein level of PINK1 is linked to γ-secretase activity and to APP but is independent of the phosphatase and tensin homolog PTEN (a regulator of PINK1 kinase activity). The study also unraveled that the modulation of the γ-secretase activity or AICD expression affects the control of mitophagy markers in a PINK1-dependent manner [41]. Interestingly, we revealed a regulatory loop in which Parkin, that transcriptionally controls PS1 and PS2 [42], acts upstream of *PINK1* to promote AICD-mediated regulation of PINK1 expression. Emerging data suggest that the early accumulation of C99, rather than that of Aβ, correlates with autophagy and mitophagy defects in AD. Accordingly, C99 accumulation was found to occur in several AD transgenic mice models and in APP KI mice harboring single or several APP FAD mutations [43]. Importantly, a recent study reported that C99 accumulation correlates with neuronal vulnerability in human AD-affected brains [44]. Studies by our group and others demonstrated that C99 accumulation induces impaired lysosomal-autophagic functions, independently of Aβ [45]. This adverse effect appears to be due to the aggregation of C99 within the endosomal-autophagic-lysosomal-vesicle membranes likely contributing to synaptic dysfunction and behavioral deficits [45–47]. C99-mediated endolysosomal dysfunctions have also been reported in human iPSC harboring AD-related *APP* or *PS1* mutations displaying endogenous APP level expression [48, 49], thus indicating that impaired lysosomal-autophagic functions is not an artifact due to an overload of mutated APP or C99 fragment in overexpressing models. In addition, our laboratory and others delineated the processing of APP in mitochondria associated membranes (MAMs) and the accumulation of APP-derived fragments in this microdomain impacting MAMs function [50–52]. Of most interest, MAMs have been described as an important hotspot for mitophagy [53]. Upon mitophagy induction, PINK1 and Bcl-1 re-localize in MAMs to promote phagosome formation [54]. Next, Parkin-dependent and PINK1 phospho-ubiquitination of the ER-mitochondria tether MFN2 uncouples mitochondria and permits its engulfment and mitophagic degradation [55]. It is noteworthy that an increase number of ER and mitochondria contact sites was reported in AD study models and human brains [50, 56]. In a recent study, we reported that APP-CTFs accumulate in the mitochondria-enriched fraction of human neuroblastoma cells expressing human APP harboring the Swedish familial mutation (SH-SY5Y-APPswe) that promotes Aβ and APP-CTFs production [18]. We first unraveled mitochondrial structure (i.e. cristae disorganization) and function alterations in these cells and reported an activation of the first steps of mitophagy characterized by increased levels of PINK1, Parkin, and LC3-II/I ratio. However, we observed that the overall mitophagy process was blocked as revealed by the lack of p62 degradation, the accumulation of several mitochondrial proteins (TIMM23, TOMM20, HSP60, and HSP10) and by impaired mitophagosome-lysosome fusion. Importantly, mitochondria structure alteration, ROSmit production and mitophagy failure phenotypes were exacerbated by the γ-secretase inhibition (that blocks Aβ production and potentiates APP-CTFs (C99 and C83) accumulation). On the contrary, β-secretase inhibition (that prevents both Aβ and C99 production) tends to ameliorate mitochondria structure and to rescue complex I activity defect and ∆Ψmit depolarization. We further confirmed the role of APP-CTFs on ROSmit overproduction and mitophagy failure in SH-SY5Y cells expressing C99 fragment only. In addition, we reported that APP-CTFs accumulate in neuronal mitochondria of the 3xTgAD mice, before any detection of amyloid plaques, as well as in adeno-associated virus C99 (AAV-C99) injected mice. Of most interest, the inhibition of the γ-secretase in pre-symptomatic 3xTgAD mice exacerbated mitochondrial structure alterations and hampered mitophagy process. These findings were recently confirmed in iNSC familial AD cell line (PS1 C737A) [57]. Compared to WT-iNSC non-isogenic controls, AD-iNSC exhibit mitochondrial accumulation of APP-CTFs, mitochondrial dysfunctions and mitophagy failure. Interestingly, the pharmacological inhibition of the γ-secretase in AD-iNSC also worsened the mitochondrial and mitophagy dysfunctions, similarly to the effect of the PS1/PS2 double knockout-iNSC [57]. Altogether, these results firmly demonstrated the contribution of APP-CTFs, independently of Aβ, to mitophagy defects in AD [18] (Fig. 2D and Table 2).

### APOE4

APOE is a glycoprotein mainly expressed in astrocytes, microglia, neurons and in the choroid plexus. APOE acts as a lipid transporter, notably for cholesterol and cholesterol esters. *APOE* is a polymorphic gene encoding for three major APOE epsilon (ε) isoforms with differential binding with lipids. It is now established that APOEε2 confers a decreased risk of AD in comparison with the APOEε3 allele. Conversely, a single APOε4 allele increases the risk of AD four-fold compared to the common APOEε3/ε3 genotype while the presence of two APOEε4/ε4 alleles augments the risk of AD by approximately twelve-folds. The impact of APOE4 on mitophagy was thoroughly scrutinized in recent years. Comparison of APOE genotypes indicated a larger accumulation of mitochondrial and aggregated proteins in glioblastoma T98G cells expressing APOE4 vs APOE3 [58]. This has been linked to a blockade of autophagy and mitophagy processes likely due to the binding of APOE4 to CLEAR (coordinated lysosomal expression and regulation) DNA motifs, thus competing with TFEB and compromising TFEB-mediated transcriptional up-regulation of *p62/SQSTM1*, *MAP1LC3B*, and *LAMP2* genes [58]. Comparative transcriptomic analyses of human post-mortem brains indeed revealed that APOEε2/ε3 carriers, compared to APOEε3/ε4 and APOEε4/ε4 carriers, displayed upregulated *MAP1LC3B*, *p62/SQSTM1*, *NBR1*, *OPTN*, and *BNIP3* transcription [59]. Accordingly, APOE4 mice display higher mitochondria markers levels (TOMM40 and COX1) and reduced cristae density in hippocampal neurons as compared to APOE3 mice [60]. These observations suggest a diminished mitophagic capacity and an accumulation of damaged mitochondria linked to APOE4 expression. The investigation of the underlying molecular mechanisms further indicated an initiation of mitophagy reflected by a reduction of cleaved PINK1, a sign of reduced ∆Ψmit, and by elevated Parkin levels in APOE4 mice [60]. However, as reported in other APOE study models, the mitophagy appears to be blocked in later steps, as evidenced by increased p62 and mitochondria markers [60]. A recent study reported that APOE4 expressing astrocytes showed impaired mitochondrial function and exhibited reduced fission and mitophagy characterized by reduced active Parkin and LC3-II proteins, accumulated p62 and by defective ubiquitination and proteasomal/lysosomal degradation of mitochondrial dynamic proteins [61]. Finally, iPSC-derived cortical neurons derived from APOEε4/ε4 SAD patients show reduced phosphorylation of mitophagy initiators TBK1 and ULK1 as well as reduced expression of PINK1, LC3-II and Bcl-1 as compared to control neurons [16]. These observations may suggest a blockade of mitophagy at the initial steps of the process. In addition, it should be noted that the APOEε4 SAD risk factor is also associated with increased cholesterol plasma levels, atherosclerosis, and oversupply of cholesterol to neurons [62]. Strikingly, it was recently described that increased intracellular cholesterol content could, by itself, impact mitophagy [63]. Indeed, acute enrichment of cholesterol in neuroblastoma SH-SY5Y cells impairs the mitophagy flux by hampering mitochondria delivery to lysosomes, even in the presence of oAβ_1–42_ or of the mitochondrial uncoupler CCCP [63]. Accordingly, chronic cholesterol accumulation in aged mice also impairs mitophagosome formation by reducing OPTN mitochondrial translocation [63] (Fig. 2E and Table 2).

## Mitophagy stimulation in ad study models and potential clinical applications

### Genetic strategies

Several genetic approaches targeting components of the mitophagy machinery have been used to alleviate toxic insults linked to the accumulation of disease-associated dysfunctional mitochondria. The overexpression of Parkin through the use of lentivectors in SAD fibroblasts improves autophagy flux and greatly favors the degradation of accumulating mitochondria [12]. Parkin overexpression also ameliorates mitochondrial functions through a recovery of ΔΨmit [12]. In another report, Parkin overexpression in Aβ-treated HEK293T cells, was able to increase cytosolic and translocated Parkin levels, mitochondrial LC3-II level, and to reduce mitochondrial p62 levels, globally indicating a rescue of mitophagy in these cells. Parkin overexpression also attenuated Aβ-induced mitochondrial fragmentation and dysfunctions as evidenced by the elevation of ΔΨmit, of the activities of complex I, II and IV, and of ATP production and by lowering ROSmit production [36]. Other studies investigated the impact of PINK1 in a preclinical AD model, showing that intra-hippocampal stereotaxic injections of AAV2-hPINK1 in 6-month-old transgenic mice overexpressing hAPP bearing the Swedish and the Indiana (V717F) mutations triggered an induction of mitophagy via an upregulation of OPTN and NDP52 mitophagic receptors [13]. Importantly, PINK1-injected mice harbored reduced Aβ pathology (number of plaques and mitochondrial Aβ accumulation) in the hippocampi and show normal mitochondrial function, reduced synaptic loss as well as improved synaptic function and learning memory [13]. DISC1 protein is known to regulate anterograde and retrograde axonal mitochondria transports. But its newly discovered function as a mitophagy receptor via a LIR-motif-dependent binding of LC3 further support the importance of DISC1 in mitophagy [19]. In vitro, DISC1 siRNA inhibited oAβ_1–42_- or CCCP-induced mitophagy, while DISC1 overexpression triggered an LC3-dependent mitophagy and rescued oAβ_1–42_-induced mitochondrial and synaptic defects [19]. Supporting these in vitro findings, DISC1 overexpression in APPswe/PS1ΔE9 (APP/PS1) mice reduced amyloid plaque density, synaptic loss and cognitive defects, through the promotion of mitophagy [19]. In neurons, the adaptor protein Snapin participates, with the motor protein Dynein, in the retrograde transport of distant mitophagosome to the soma, to complete mitophagic degradation [38]. Deficiency of Snapin in wild type murine brains recapitulates AD synaptic defects by abolishing Snapin-mediated retrograde transport and causing presynaptic mitophagic stress [38, 64]. On the contrary, Snapin overexpression diminished the presynaptic mitochondria stress and synapses loss in hAPPswe/ind AD mice, by facilitating the retrograde transport of axonal mitophagosomes [38, 64]. Finally, the overexpression of Miro1 (a mitochondrial Rho GTPase implicated in mitochondrial anterograde and retrograde transport) [65] rescued damaged mitochondrial morphology and motility and mitigated oAβ-mediated mitophagy [66].

### Pharmacological strategies

_Urolithin A (UA), derived from ellagitannins polyphenols, is known to induce beneficial effects on mitochondrial homeostasis and functions through the stimulation of mitophagy in nematodes and mammals [67]. Recently, Fang et al. studied the impact of UA in various in vivo AD models and described significant improvements of pathological parameters via an induction of mitophagy [16]. Hence, the treatment of *C. elegans* expressing Aβ_1–42_ and of APP/PS1 mice with UA reduced Aβ pathology and cognitive decline, via the activation of PINK1/Parkin-dependent mitophagy. UA was also shown to rescue mitochondria structural and functional defects and to increase synapse number. Further, UA decreased mitochondrial damage in microglia, stimulated the phagocytic clearance of Aβ plaques and reversed inflammatory responses. UA treatment also enhanced memory and reduced Tau hyperphosphorylation in the 3xTgAD mice [16]. The first human clinical trial of UA (500 mg and 1000 mg for 4 weeks) in healthy sedentary elderly individuals demonstrated its safety and its benefit by modulating mitochondrial gene expression (NCT02655393) [68].

_Actinonin (AC) is a natural antibacterial compound showing similar effects than UA [16]. AC treatment was able to avert memory defects and to reduce APP-CTFs load and Aβ burden in an AD nematode model. In APP/PS1 mice, AC also restored mitochondria morphology and functions and enhanced synapse number by stimulating mitophagy. Interestingly, AC promoted mitophagy in microglia by stimulating Aβ plaque clearance and reducing neuro-inflammation.

_NAD^+^ boosters (nicotinamide riboside (NR), nicotinamide mononucleotide (NMN)) are robust inducers of mitophagy [69]. NAD^+^ is a cofactor for several proteins, including Sirtuins (SIRT1, 3, 6 and 7), able to stimulate general autophagy and mitophagy through different pathways [70]. In *C. elegans* expressing Aβ_1–42_, NR induced mitophagy, reduced Aβ burden and proteotoxic stress, and boosted health and lifespan [71] while NMN enhanced PINK1/Parkin-dependent mitophagy and reverses memory defects in this model [16]. NR also reduced cortical Aβ deposits, enhanced PINK1, LC3, and OXPHOS protein mRNA levels, and improved cognitive functions in APP/PS1 mice [71]. Several clinical trials are ongoing to assess the effect of NR on brain function, cognition, oxidative stress, or CSF pTau levels in MCI (mild cognitive impairment) and AD patients (NCT02942888, NCT04078178, NCT04430517, NCT03482167 and NCT03061474). However, Nicotinamide failed to improve cognitive functions in a small clinical trial of patients with mild to moderate AD [72].

_Trehalose is a natural disaccharide acting as an mTOR-independent autophagy inducer, and a TFEB and lysosomal activator. It protects mitochondria from oxidative stress via a BNIP3-induced mitophagy [73, 74]. Trehalose administration in vivo showed neuroprotective benefits in different AD mouse models [75].

_Resveratrol (3,5,4′-trihydroxy-*trans*-stilbene), a natural polyphenolic molecule acting as a ROS scavenger, an iron chelator and an autophagy and mitophagy inducer [76]. It displays several beneficial effects in the context of AD [76]. Resveratrol protects PC12 cells from death, oxidative stress and mitochondrial damage induced by Aβ_1–42_ by promoting mitophagy [34]. Long-term oral administration of resveratrol in APP/PS1 mice improved memory as well as mitochondrial functions, activated SIRT1 and AMPK pathways and reduced Aβ load [77]. Nevertheless, this molecule has a major clinical drawback, since it is metabolically unstable and thus offers a poor bioavailability [78].

_Bexarotene is a synthetic molecule used as a retinoic X receptor agonist. Bexarotene enhanced Aβ clearance, improved cognitive deficits in APP/PS1 mice [79], and reduced Tau levels in the CSF and the memory deficit in a patient with mild AD [80]. Recently, Martín-Maestro et al. demonstrated that Bexarotene stimulated autophagy and PINK1/Parkin-dependent mitophagy in FAD PS1 (M146L) NSC, thus allowing the elimination of dysfunctional mitochondria and restoring the mitochondrial network morphology [24].

_Tetrahydroxy stilbene glycoside is extracted from the traditional Chinese medicinal herb *Polygonum multiflorum* and was described to harbor neuroprotective effects in AD [81]. This beneficial effect seems to occur by targeting autophagy and mitophagy through the AMPK/PINK1/Parkin cascade [82].

_β-Asarone, the main effector of *Acorus tatarinowii* Schott herbal medicine activated autophagy and mitophagy and alleviated Aβ_1-42_ cytotoxicity in vitro [33]. Studies in vivo reported that β-Asarone had beneficial effects against Aβ-related pathology through the inhibition of autophagy in APP/PS1 mice [83] and decreased Aβ_1–42_ brain levels. Another study rather described that β-Asarone improved learning and memory in rats injected with Aβ_1-42_ by promoting mitophagy [37].

_The circadian hormone melatonin, mostly holds several physiological roles among which neuroprotection. Sun et al. recently reported that long-term oral administration of melatonin in PS1 mice had a neuroprotective action by improving mitochondrial structure and mitigating the excessive mitophagy through a reduction of the expression of mitophagy proteins (PINK1, Parkin, LC3-II/LC3-I) and of the number of mitophagic vesicles [84]. Melatonin treatment also down-regulated APP processing and improved spatial learning and memory defects [84].

_Spermidine is a small and natural organic molecule known to prolong lifespan of yeast, flies, nematodes and mice by upregulating autophagy [85, 86]. It also stimulates the PINK1/Parkin mitophagy pathway in human fibroblasts [87]. Interestingly, it has recently been documented that spermidine extended the lifespan and prevented memory loss in a PINK1/Parkin-pathway-dependent fashion in *C. elegans* expressing human Aβ and Tau [88]. In humans, a clinical trial in elderly subjects with MCI, concluded that spermidine may improve hippocampal-dependent memory (NCT02755246) [89].

_UMI-77 is a small BH3-mimetic recently identified in a large screen as mitophagy inducer targeting the mitophagy receptor MCL-1 [90]. UMI-77 normalized mitochondrial morphology and induced mitophagy in APP/PS1 mice [90] triggering a rescue of learning and memory capacities, a reduction of the amyloid pathology and of neuroinflammation [90].

_Kaempferol (flavonoid) and Rhapontigenin (stilbenoid) are two natural compounds recently identified as mitophagy inducers by a high-throughput machine-learning screen, and validated in vitro and in vivo [91]. These two drugs reduced the levels of pTau, APP-CTFs and the Aβ load in cells, worm, or mouse models of AD. Kaempferol and Rhapontigenin restored memory deficits in both Tau and Aβ AD study models [91]. These beneficial effects were due to a stimulation of mitophagy via an upregulation of several mitophagy actors and the promotion of mitochondrial fission [91]. Of the two compounds, Kaempferol is already known to be able to cross the blood-brain barrier (BBB) [92] and may reduce the risk of developing AD [93].

_Metformin (MET), derived from the gelegine of the medicinal plant *Galega officinalis*, is a proven anti-hyperglycemic drug used in the context of Type 2 diabetes mellitus (T2DM). MET promotes mitophagy via the activation of AMPK in vivo and in humans [94–96]. It was demonstrated that MET stimulated the autophagic clearance of pTau in vitro in HT22 cells incubated with high glucose and in vivo in the hippocampus of diabetic mice, thereby rescuing their cognitive impairments [97]. In AD preclinical studies, MET reduced Tau phosphorylation in murine primary neurons expressing hTau via the activation of the PP2A phosphatase [98], and prevented amyloid plaques formation, cognitive impairment and chronic neuroinflammation in APP/PS1 mice [99]. A short clinical trial (8 weeks) in AD patients with a high-dose oral administration of MET demonstrated both the presence of MET in the CSF, and the improvement of executive functions, learning, memory and attention (NCT01965756) [100]. A Phase II/III prevention trial, called Metformin in Alzheimer’s Dementia Prevention, is ongoing and includes 370 AD patients treated with 2000 mg of MET per day or with a placebo for 2 years (NCT04098666). T2DM is considered a risk factor of developing SAD [101]. Of most interest, T2DM patients treated with MET show a lower risk of dementia compared to untreated T2DM patients [102]. Accordingly, a meta-analysis also concluded that the incidence of both dementia and cognitive impairments were significantly lower in MET-treated diabetics [103].

### Lifestyle strategies

Physical exercise (PE) is already used in the context of several metabolic or mental disorders and has been reported to be beneficial in the context of AD [104], even though the precise mechanism remained unknown. In AD mouse models, PE provided beneficial effects on synaptic plasticity alterations, restored neuroprotective factor levels, improved cognitive deficits and reduced Aβ production. Several studies converged to propose that PE acted through the SIRT1/PINK1/Parkin signaling pathway [105–107]. Moreover, in APP/PS1 mice, PE increased mitophagy and ameliorated learning and memory capacities, Aβ burden, synaptic activity, and mitochondrial structural and functional alterations [108]. Caloric restriction (CR) is a strong activator of mitophagy [109]. In AD mice models, CR up-regulated SIRT1 and NAD^+^ levels [110], reduced hippocampal Aβ and Tau load [111, 112] and ameliorated behavioral deficits [112].

## Conclusion

This review summarized up-to-date studies linking mitophagy failure to AD pathogenesis. Data in human post-mortem brains and derived cells highlighted that mitophagy failure is common to both SAD and FAD cases. Studies in preclinical AD models, convincingly demonstrated that several AD actors (Tau, Aβ, APP-CTFs, APOE4, PS1 mutants) impair the degradation of dysfunctional mitochondria. Genetic targeting of deficient mitochondrial mitophagic degradation by overexpressing proteins implicated at different steps of this process resulted in a significant rescue of several AD stigmata. These consensual results supported the idea that rescuing mitophagy in AD could be an effective therapeutic strategy. In fact, several molecules able to stimulate mitophagy have shown positive effects in preclinical AD models. Some of these drugs have also been administrated to healthy or AD patients at various stages in clinical trials (UA, NR, NMN, spermidine, and MET) [113]. However, there is still a need to optimize these drug candidates and particularly their bioavailability (i.e. biodegradation, crossing of the BBB), and their pharmacokinetics and interaction with their targets at the relevant site. To improve the traditional time-consuming approaches for drug discovery, researchers have now access to machine learning-based virtual screening combined with cross-species platform-supported wet lab validation [91]. According to the mitochondrial cascade hypothesis, mitochondrial dysfunctions are primary events in AD disease contributing to downstream pathological molecular pathways accelerating disease progression [114]. Thus, is it conceivable to stimulate mitophagy in healthy or at-risk persons to prevent AD development? Will a single mitophagy stimulating molecule be effective enough? Or, should we envision the administration of mitophagy stimulating drugs in combination with PE or CR lifestyle changes, or even in combination with well-known in-test strategies (i.e., monoclonal antibodies against Aβ_1-42_, pyroglutamate Aβ or pTau species)?
